# What is Unique About Kindness? Exploring the Proximal Experience of Prosocial Acts Relative to Other Positive Behaviors

**DOI:** 10.1007/s42761-022-00143-4

**Published:** 2022-10-07

**Authors:** Annie Regan, Seth Margolis, Daniel J. Ozer, Eric Schwitzgebel, Sonja Lyubomirsky

**Affiliations:** University of California, Riverside, Riverside, CA USA

**Keywords:** Prosocial behavior, Social connection, Eudaimonia, Well-being

## Abstract

Previous research has identified a robust connection between prosociality and happiness, suggesting that kindness has both hedonic and eudaimonic benefits—in the short term and in the long term. By contrast, our experiment aimed to examine people’s momentary eudaimonic feelings *while* engaging in kind acts for others. To that end, we randomly assigned participants to one of four positively valenced conditions that varied in their inclusion of potential “active ingredients” of prosocial behavior. Namely, engaging in kind acts for others was compared to engaging in kind acts for oneself (social element removed), extraverted behavior (kindness element removed), and open-minded behavior (both social and kindness elements removed). Participants were assessed five times over 2 weeks, each time reporting on how they felt during their assigned activities. Multilevel models revealed that relative to all other conditions, participants assigned to do kind acts for others reported a greater sense of competence, self-confidence, and meaning while engaging in those acts across the intervention period. Engaging in acts of kindness for others also led to stronger feelings of connection relative to engaging in open-minded behavior or acts of kindness for oneself but did not differ from engaging in extraverted behavior. These results illuminate the experience of positive eudaimonic feelings while one commits kind acts for others and highlight the unique benefits of prosociality compared to other positive behaviors.

**Supplementary Information:**

The online version contains supplementary material available at 10.1007/s42761-022-00143-4.

Humans are a markedly prosocial (i.e., kind) species, and the benefits of prosocial behavior are well-established. Our focus here, however, is not on the hedonic benefits of kindness but on its momentary experience from the actor’s perspective. Accordingly, this study seeks to investigate the unique, subjective experience of acting prosocially, in an effort to understand what sets it apart from other positive behaviors.

## Definitions of Well-being

Before reviewing the benefits of prosocial behavior for well-being, we first acknowledge that psychological well-being is a broad construct with multiple definitions and corresponding measures. According to one of the most widely used definitions, subjective well-being (SWB) comprises two components: a cognitive component, which involves the individual’s judgments about their life, and an affective component, which involves the individual’s experience of positive and negative affect (Diener, 1984; see Diener et al., 2018, for a review). SWB is often contrasted with other conceptualizations of well-being, including eudaimonic well-being, which is characterized by a sense of meaning in life, fulfilling relationships, and sense of self-efficacy (Ryff, 1989; Waterman, 2013). Recent work has sought to integrate these two constructs, situating eudaimonia as a class of activities, or “well-doing,” which satisfy key psychological needs and, in turn, increase SWB (Martela & Sheldon, 2019; Sheldon, 2018). The present study, which focuses on participants’ subjective experience of engaging in prosocial behavior relative to other types of positive behavior, takes the latter approach to eudaimonia.

## Prosociality and Well-being

Although prosocial behavior is enacted to benefit others, numerous studies have shown that it benefits the actor in addition to its intended recipient (see Hui et al., 2020, for a meta-analysis). For example, adults who spend time volunteering report lower levels of depression and greater happiness than non-volunteers (Borgonovi, 2008; Musick & Wilson, 2003). Experimental work has demonstrated a causal relationship between prosociality and well-being (see Curry et al., 2018, for a meta-analysis). Those instructed to spend money on others (i.e., prosocial spending), for example, report greater happiness relative to those instructed to spend money on themselves (Aknin et al., 2020; Dunn et al., 2008). Further, individuals assigned to act more prosocially over several weeks report greater subjective happiness, life satisfaction, and flourishing mental health than those assigned to control activities—including engaging in acts of kindness for oneself (Chancellor et al., 2018; Nelson et al., 2015, 2016). In other words, acting prosocially *feels* good. Such studies suggest a potential explanatory mechanism—namely, hedonic rewards—for how prosocial behavior may be encouraged and maintained.

The work described above largely represents the global SWB benefits of prosocial behavior, or how an individual thinks and feels about their life. Prosociality may have eudaimonic rewards as well, as it has been shown to satisfy the actor’s key psychological needs—namely, autonomy, competence, and relatedness (Ryan & Deci, 2000; Weinstein & Ryan, 2010). Indeed, experimental work has shown that being prosocial may lead people to feel more competent, more in control, and more connected (Martela & Ryan, 2016; Nelson et al., 2015; Titova & Sheldon, 2021). Thus, prosocial behavior satisfies the actor’s needs at the same time as the actor is meeting or attending to the needs of others. Accordingly, being kind has been shown to be a meaningful experience (Van Tongeren et al., 2016). For example, participants instructed to spend money on others rather than themselves reported greater meaning in life, and this relationship was mediated by perceptions of self-worth (Klein, 2017). In sum, research suggests that engaging in prosocial behavior is a psychologically rich experience, but no experiments to our knowledge have simultaneously explored the unique feelings elicited by prosocial behavior compared to multiple alternative positive activities.

## Present Study

We posit that investigating the proximal experience of kindness can illuminate phenomenological differences between kindness and other types of positive, socially desirable activities. To that end, we randomly assigned participants to engage in one of four positive behaviors over the course of 15 days. We hypothesized that participants who engaged in prosocial behavior would report stronger momentary eudaimonic feelings than those who completed other positive activities.

To test our hypothesis, we assigned participants to engage in different types of positive activities, varying in their inclusion of key components of prosocial behavior. Prosocial behavior is typically social, generous, and positive. Accordingly, engaging in kind acts for others was compared to engaging in kind acts for oneself (social element removed), extraverted behavior (kindness element removed), and open-minded behavior (both social and kindness elements removed). Although prosocial behavior has been contrasted with self-focused and extraverted behavior in prior experimental research (Aknin et al., 2020; Dunn et al., 2008; Fritz et al., 2022; Nelson et al., 2015), the present study is novel in its inclusion of multiple positively valenced comparison conditions and is the first experiment to our knowledge to compare prosocial behavior to open-minded behavior.

## Method

### Participants

Participants included 671 Australian adults (age 18 to 84; *M*_age_ = 45.35, *SD* = 15.78) recruited from PureProfile, an online panel company. The majority of participants identified as female (67.2%) and White/Caucasian (83.9%), followed by Asian (7.3%), and other or multiple ethnicities (8.8%). The sample size per condition was as follows: Acts of Kindness for Others (*n* = 158), Acts of Kindness for Self (*n* = 182), Extraverted Behavior (*n* = 166), and Open-Minded Behavior (*n* = 165).

### Procedure

**Fig. 1:**
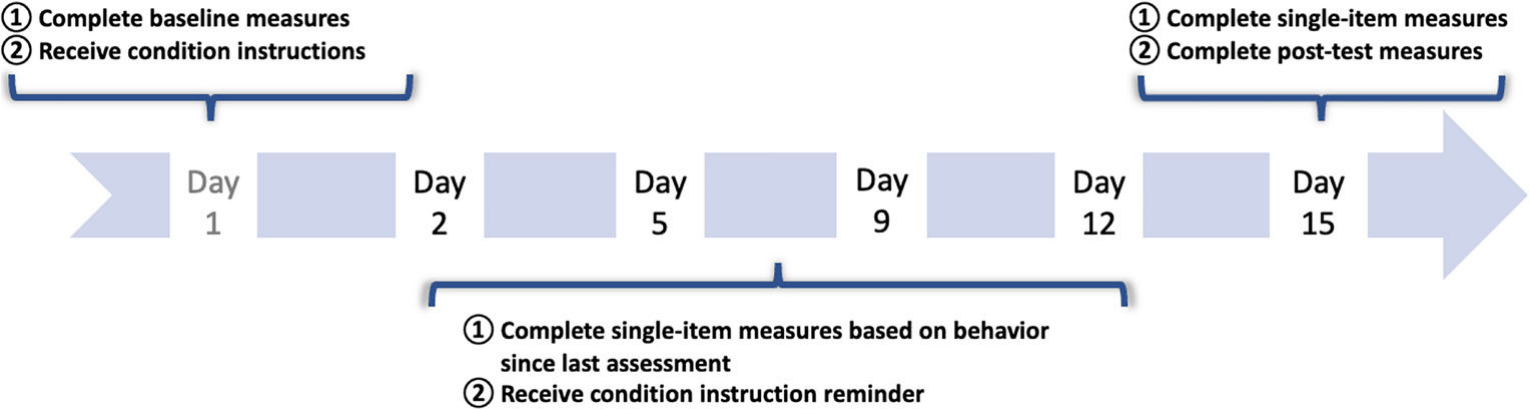
Study timeline. *Note*: Complete study timeline. The present analyses focus on participants’ experience while engaging in their assigned behaviors and thus only includes the single-item measures completed on days 2, 9, 12, and 15

All intervention instructions and assessments were administered via online surveys within the PureProfile platform. Upon completion of a baseline survey, participants were randomly assigned to one of four intervention conditions, in which they were instructed to engage in acts of kindness for themselves, acts of kindness for others, extraverted behavior, or open-minded behavior over the course of 15 days (see Fig. 1 for study timeline). Participants were given three examples of their assigned behavior to facilitate adherence with the intervention instructions. For example, those instructed to engage in acts of kindness for themselves were given the example of “Treating yourself to a massage,” and those instructed to act more open-minded were given the example of “Engage more with art, music, or literature” (see Table 1 for abridged condition instructions and Supplemental Materials for complete condition instructions). To facilitate behavior change, participants were asked to create five specific plans for when and where they planned to incorporate the assigned activity into their daily lives during the subsequent 2 weeks (similar to the instructions used by Hudson & Fraley, 2015; cf. Gollwitzer & Brandstätter, 1997). Participants received their plans via text message and email for reference.

**Table 1:** Study design and condition instructions

Condition	Characteristics (“ingredients”) of behavior	Instructions
Acts of kindness for others (*n* = 158)	Kind Social Positive/socially desirable	During the next 2 weeks, we would like you to try to acts of kindness for others. Here are some example activities to give you a better idea of what we mean by “acts of kindness for others”: • Cooking dinner for friends or family • Doing a chore for a family member • Paying for someone’s coffee in line behind you
Acts of kindness for self (*n* = 182)	Kind Positive/socially desirable	During the next 2 weeks, we would like you to try to do acts of kindness for yourself. Here are some example activities to give you a better idea of what we mean by “acts of kindness for yourself”: • Enjoying a day trip • Treating yourself to a massage • Having your favorite meal
Extraverted behavior (*n* = 166)	Social Positive/socially desirable	During the next 2 weeks, we would like you to try to act as extraverted as you can. Here are some example activities to give you a better idea of what we mean by “extraverted”: • Spend more time with people • Speak up more when people say things you disagree with • Engage in more stimulating activities When doing these activities, try not to interact with people you already know and instead try to engage with people you don’t know very well (e.g., strangers).
Open-minded behavior (*n* = 165)	Positive/socially desirable	During the next 2 weeks, we would like you to try to act as open-minded as you can. Here are some example activities to give you a better idea of what we mean by “open-minded”: • Engage more with art, music, or literature • Do more to pursue an intellectual curiosity • Think more creatively, either throughout your daily life or by setting aside time to think about things in a creative manner When doing these activities, try not to interact with people and instead try to engage with your own thoughts.

Abridged condition instructions and sample behaviors. See Supplemental Materials for complete condition instructions and change plan prompts

After the baseline assessment, participants received two additional surveys per week for the duration of the study, for a total of six measurement occasions (baseline [day 1], four intermediary assessments [days 2–12], and posttest [day 15]). Specifically, participants received a Qualtrics survey from PureProfile one day after they completed the baseline assessment (day 2), then on days 5, 9, 12, and 15. Surveys were completed in April and May of 2018.

To capture participants’ feelings while engaging in the four types of positive activities, we first asked participants to reflect on the ways in which they changed their behavior as a result of the intervention and then respond to single-item self-report measures based on how they felt *while engaging in* each behavior they completed since the last assessment. Participants were able to report on their feelings about up to three behaviors per measurement occasion. Participants also completed a longer battery of measures at baseline (day 1) and posttest (day 15), including measures of positive and negative affect, life satisfaction, and personality traits. The present study, however, focused on participants’ proximal (or immediate) experience, using only the single-item measures completed after participants engaged in their assigned behaviors (days 2, 5, 9, 12, and 15). The analyses included in this report include all measures delivered at the intermediary assessments. Results of the pre-test to post-test analyses for global well-being outcomes will be reported elsewhere. This study was approved by our university’s institutional review board.

## Measures

Eudaimonia Eudaimonic feelings were assessed using 6 single items to assess participants’ sense of meaning (“I felt a sense of purpose or meaning”); self-confidence (“I felt confident about my abilities”); energy level (“I felt like I was full of energy”); and psychological needs (Ryan & Deci, 2000; adapted from the Balanced Measure of Psychological Needs [BMPN]; Sheldon & Hilpert, 2012), including their sense of autonomy (“I felt free to do things my own way”), connectedness (“I felt close and connected with other people”), and competence (“I felt very capable in what I was doing”). All items were rated on a 7-point Likert scale ranging from 1 (*strongly disagree*) to 7 (*strongly agree*). To ensure participants were reporting how they felt while engaging in their assigned behavior, all items were preceded by the following prompt: “Think of the [first, second, third] act you did for this study after the last multi-question assessment. Please rate your agreement with the following statements about how you felt during that act.”

We created a eudaimonia composite variable of all single-item measures. Given the nested structure of our data, we calculated multilevel reliability for this composite variable using the multilevel reliability function in the psych R package (Revelle & Condon, 2019; Revelle & Wilt, 2019; cf. Shrout & Lane, 2012). The reliability of all ratings across all timepoints (i.e., reliability of fixed effects; *R*_kF_) and the generalizability of between-person differences averaged over time (i.e., time nested within subjects; *R*_kRn_) were good (*R*_kF_ = .97; *R*_kRn_ = .85). The generalizability of within-person variation averaged over items (i.e., time nested within subjects) was moderate (*R*_cn_ = 0.77).

Socially Desirable Responding We measured socially desirable responding with the 16-item Balanced Inventory of Desirable Responding (BIDR-16; Hart et al., 2015). The BIDR-16 measures two dimensions of socially desirable responding—impression management (e.g., “When I hear people talking privately, I avoid listening”) and self-deceptive enhancement (e.g., “I never regret my decisions”). Participants rated items on these two subscales on a 7-point scale (1 = *strongly disagree,* 7 = *strongly agree*). Participants completed this measure at baseline (Impression Management Subscale *α*= .76; Self-Deceptive Enhancement Subscale *α*= .75).

Experimenter Demand To assess and account for the extent to which our effects were driven by demand created by the experimental prompt, we used the 4-item Perceived Awareness of the Research Hypotheses Scale (Rubin, 2016). Participants rated their agreement with items such as “I had a good idea about what the hypotheses were in this research” on a scale from 1 (*strongly disagree*) to 7 (*strongly agree*). Participants completed this measure on day 15 (*α*= .87).

### Analytic Approach

To account for the nested structure of our data (assessments nested within days nested within participants), we fit three-level multilevel models for all outcomes. Analyses were conducted using the lme4 and lmerTest R packages. Participant and participant × day number (i.e., time) were included as random intercepts in all models, and slopes were fixed. Condition was dummy coded such that acts of kindness for others served as the reference group (coded 0 on all pseudovariates) for each of the other conditions in all models. The three pseudovariates were then each multiplied by −1 so that a positive fixed effect coefficient corresponded to the degree to which the acts of kindness for others condition was relatively *higher* than each of the other conditions. Given the positively toned experimental instructions for all four conditions, measures of socially desirable responding and experimenter demand were included as level 3 covariates in all models. We included act number (the order in which participants reported their specific behaviors) and time (day number) as level 1 and 2 (respectively) fixed effect covariates in all models to ensure our effects were not simply due to the passage of time or order of participants’ reported acts. Partial *r* coefficients were computed for all fixed-effects using the *t*-value for each coefficient and the degrees of freedom estimated from the Satterthwaite approximation (generated from the lmerTest package in R).

## Results

**Fig. 2:**
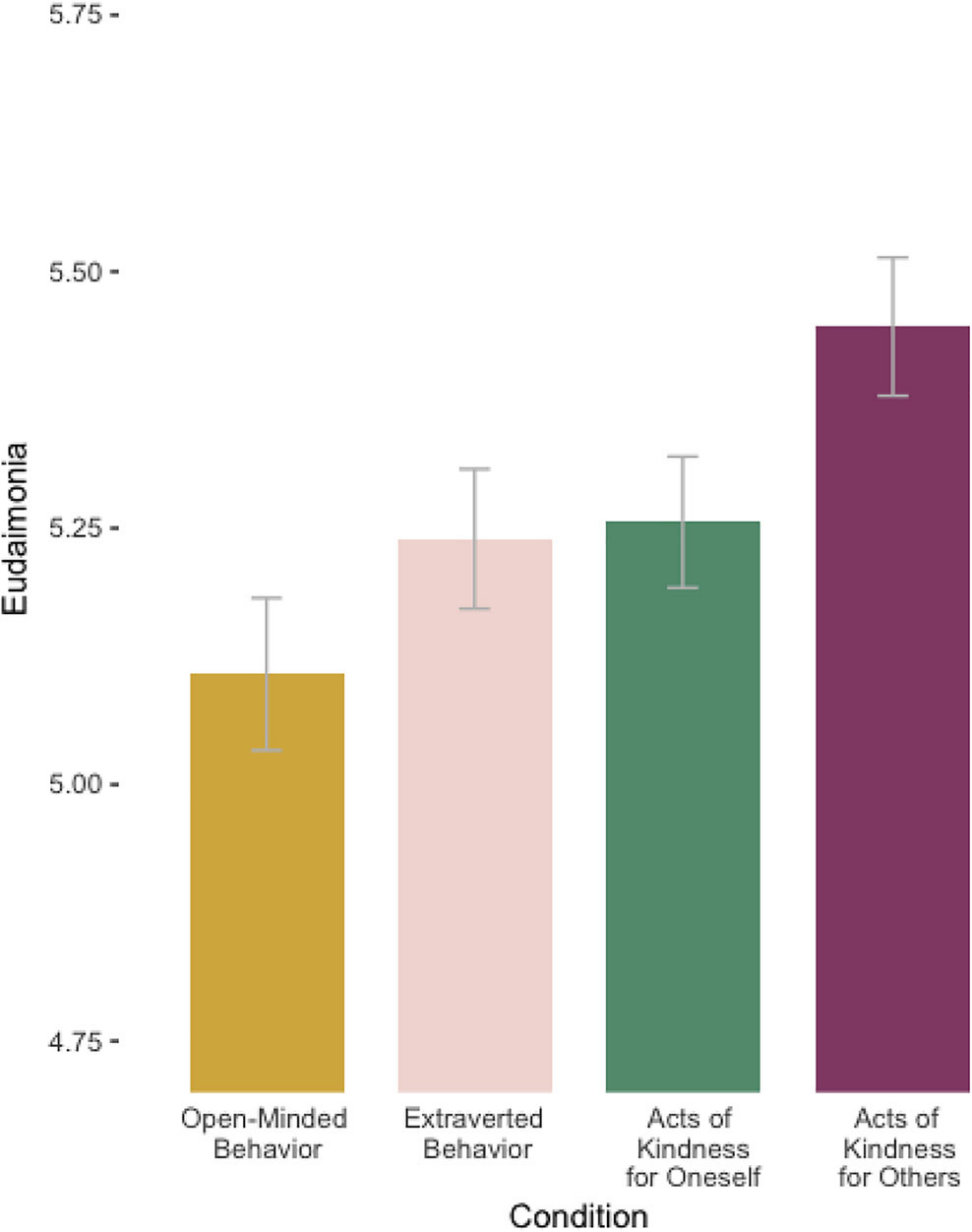
Condition means: Condition means are collapsed across the intervention period to represent overall differences among groups

Means and standard deviations for all outcomes are presented in Table 2, and unstandardized coefficients and partial *r*s for fixed effects are included in Table 3 (see Tables S1–S8 in Supplemental Materials for complete model summaries; see Tables S9–S16 for unconditional models). As predicted for the eudaimonia composite variable, those who engaged in acts of kindness for others reported greater eudaimonic feelings than those who engaged in open-minded behavior or acts of kindness for themselves (see Fig. 2). Participants who engaged in acts of kindness for others did not differ from those who engaged in extraverted behavior, however.^1^

**Table 2:** Means and standard deviations for all outcome variables

Outcome	Acts of kindness for others *n* = 158 *M* (SD)	Acts of kindness for self *n* = 182 *M* (SD)	Extraverted behavior *n* = 166 *M* (SD)	Open-minded behavior *n* = 165 *M* (SD)
Connectedness	5.27 (1.34)	4.88 (1.49)	5.13 (1.32)	4.69 (1.60)
Autonomy	5.49 (1.27)	5.43 (1.26)	5.31 (1.27)	5.30 (1.43)
Competence	5.72 (1.16)	5.47 (1.26)	5.39 (1.31)	5.35 (1.42)
Self-confidence	5.71 (1.19)	5.48 (1.27)	5.37 (1.33)	5.34 (1.41)
Meaning	5.55 (1.31)	5.32 (1.36)	5.27 (1.31)	5.25 (1.46)
Energy	4.94 (1.55)	4.95 (1.61)	4.96 (1.49)	4.73 (1.70)
Eudaimonia (composite)	5.45 (1.07)	5.26 (1.15)	5.24 (1.12)	5.11 (1.25)
Eudaimonia (excluding energy)	5.55 (1.06)	5.32 (1.13)	5.30 (1.12)	5.18 (1.24)

Because the analyses presented in this study focus on participants’ ratings of their feelings during each behavior, rather than changes in their feelings over time, we have collapsed these ratings across the entire intervention period (days 2–15) for the purpose of this table

**Table 3:** Multilevel model fixed effects

Outcome	Predictor	*b* [95% CI]	Partial *r* [95% CI]	*p*
Connectedness	Acts of kindness for self	0.37 [0.16, 0.58]	0.13 [0.06, 0.2]	.001
	Extraverted behavior	0.08 [−0.13, 0.29]	0.03 [−0.05, 0.10]	.457
	Open-minded behavior	0.52 [0.30, 0.73]	0.18 [0.11, 0.25]	< .001
	Experimenter demand	0.12 [0.06, 0.18]	0.16 [0.08, 0.23]	< .001
	Socially desirable responding	0.26 [0.18, 0.35]	0.23 [0.16, 0.30]	< .001
	Day number	0 [0, 0.01]	0.03 [−0.01, 0.07]	.164
	Act number	0.01 [−0.01, 0.03]	0.01 [−0.01, 0.04]	.329
Autonomy	Acts of kindness for self	0.04 [−0.14, 0.22]	0.02 [−0.06, 0.09]	.655
	Extraverted behavior	0.11 [−0.07, 0.29]	0.05 [−0.03, 0.12]	.239
	Open-minded behavior	0.13 [−0.05, 0.31]	0.05 [−0.02, 0.13]	.167
	Experimenter demand	0.12 [0.07, 0.17]	0.18 [0.11, 0.25]	< .001
	Socially desirable responding	0.33 [0.26, 0.40]	0.33 [0.26, 0.39]	< .001
	Day number	0 [−0.01, 0.01]	0.01 [−0.03, 0.05]	.791
	Act number	−0.02 [−0.04, 0]	−0.03 [−0.06, −0.01]	.012
Competence	Acts of kindness for self	0.23 [0.06, 0.41]	0.10 [0.02, 0.17]	.009
	Extraverted behavior	0.27 [0.09, 0.45]	0.11 [0.04, 0.19]	.003
	Open-minded behavior	0.31 [0.13, 0.49]	0.13 [0.06, 0.20]	.001
	Experimenter demand	0.10 [0.05, 0.14]	0.15 [0.08, 0.23]	< .001
	Socially desirable responding	0.39 [0.32, 0.46]	0.39 [0.33, 0.45]	< .001
	Day number	0 [−0.01, 0]	−0.01 [−0.05, 0.03]	.516
	Act number	−0.06 [−0.08, −0.05]	−0.09 [−0.12, −0.07]	< .001
Self-confidence	Acts of kindness for self	0.22 [0.04, 0.39]	0.09 [0.02, 0.16]	.018
	Extraverted behavior	0.28 [0.09, 0.46]	0.11 [0.04, 0.19]	.003
	Open-minded behavior	0.32 [0.13, 0.50]	0.13 [0.06, 0.20]	.001
	Experimenter demand	0.10 [0.05, 0.15]	0.15 [0.08, 0.22]	< .001
	Socially desirable responding	0.42 [0.34, 0.49]	0.40 [0.34, 0.46]	< .001
	Day number	0 [−0.01, 0]	−0.01 [−0.05, 0.03]	.644
	Act number	−0.05 [−0.07, −0.03]	−0.08 [−0.1, −0.05]	<.001
Meaning	Acts of kindness for self	0.21 [0.02, 0.41]	0.08 [0.01, 0.16]	.032
	Extraverted behavior	0.21 [0.01, 0.41]	0.08 [0, 0.15]	.042
	Open-minded behavior	0.23 [0.03, 0.43]	0.09 [0.01, 0.16]	.024
	Experimenter demand	0.14 [0.09, 0.19]	0.20 [0.13, 0.27]	< .001
	Socially desirable responding	0.33 [0.25, 0.41]	0.31 [0.24, 0.37]	< .001
	Day number	0 [−0.01, 0.01]	0.01 [−0.03, 0.05]	.655
	Act number	−0.04 [−0.05, −0.02]	−0.05 [−0.08, −0.03]	<.001
Energy	Acts of kindness for self	−0.04 [−0.29, 0.21]	−0.01 [−0.09, 0.06]	.744
	Extraverted behavior	−0.09 [−0.35, 0.16]	−0.03 [−0.10, 0.05]	.469
	Open-minded behavior	0.15 [−0.1, 0.41]	0.04 [−0.03, 0.12]	.248
	Experimenter demand	0.15 [0.09, 0.22]	0.17 [0.10, 0.24]	< .001
	Socially desirable responding	0.27 [0.17, 0.38]	0.20 [0.13, 0.27]	< .001
	Day number	−0.01 [−0.01, 0]	−0.04 [−0.08, 0]	.050
	Act number	0.01 [−0.01, 0.03]	0.01 [−0.01, 0.04]	.298
Eudaimonia (composite)	Acts of kindness for self	0.17 [0, 0.35]	0.07 [0, 0.15]	.053
	Extraverted behavior	0.14 [−0.04, 0.32]	0.06 [−0.02, 0.13]	.118
	Open-minded behavior	0.28 [0.10, 0.45]	0.12 [0.04, 0.19]	.002
	Experimenter demand	0.12 [0.07, 0.17]	0.19 [0.12, 0.26]	< .001
	Socially desirable responding	0.33 [0.26, 0.41]	0.34 [0.28, 0.40]	< .001
	Day number	0 [−0.01, 0]	0 [−0.04, 0.04]	.831
	Act number	−0.03 [−0.04, −0.01]	−0.06 [−0.08, −0.03]	<.001
Eudaimonia (excluding energy level)	Acts of kindness for self	0.21 [0.04, 0.38]	0.10 [0.02, 0.17]	.013
	Extraverted behavior	0.19 [0.02, 0.36]	0.08 [0.01, 0.16]	.032
	Open-minded behavior	0.30 [0.13, 0.47]	0.13 [0.06, 0.20]	.001
	Experimenter demand	0.11 [0.07, 0.16]	0.19 [0.11, 0.26]	< .001
	Socially desirable responding	0.35 [0.28, 0.42]	0.36 [0.30, 0.42]	< .001
	Day number	0 [0, 0.01]	0.01 [−0.03, 0.05]	.788
	Act number	−0.03 [−0.04, −0.02]	−0.07 [−0.1, −0.05]	<.001

Experimental condition was coded such that each group is being compared to the Acts of Kindness for Others group. The three condition pseudovariates were each multiplied by −1 so that a positive coefficient corresponded to the degree to which the acts of kindness for others condition was relatively higher than each of the other conditions

To unpack these results, we analyzed the eudaimonic items individually. Specifically, participants who engaged in acts of kindness for others were more likely to report having higher self-confidence, more competence, and a greater sense of meaning than all other conditions across the intervention period. Those who engaged in acts of kindness for others also reported greater feelings of connection than those who engaged in open-minded behavior or acts of kindness for themselves, but did not differ from those who engaged in extraverted behavior. Although socially desirable responding and experimenter demand significantly predicted all outcomes, the overall pattern of results did not change when these covariates were removed. Act number negatively predicted overall eudaimonic feelings, autonomy, competence, self-confidence, and sense of meaning. This pattern of results suggests that participants reported their most memorable behavior first at each assessment, resulting in an order effect of the reported acts across the intervention period. Finally, we did not detect differences between conditions in participants’ reported autonomy or energy levels.

## Discussion

Our results demonstrate the unique experience of engaging in prosocial behavior: Helping others led to greater momentary eudaimonic feelings than did other positive activities. First, and notably, participants prompted to do acts of kindness for others reported a stronger sense of meaning than those prompted to be more kind to themselves, more social, or more open to art or music. One possible explanation for this finding is that, unlike participants who engaged in other types of positive activities, those who performed acts of kindness may have done so to fill a particular need or address a specific concern of a friend, family member, or stranger. That is, if their prosocial acts had a clear objective or tangible impact, participants may have felt a heightened sense of meaning and purpose (Berg et al., 2013; Klein, 2017).

Despite its other-focused nature, engaging in kindness also had implications for participants’ self-regard. Those who did acts of kindness for others reported greater self-confidence and feelings of competence relative to those who did other positive behaviors—possibly because their kind acts were meeting another person’s needs. Additionally, unlike the behaviors assigned in other conditions, prosocial acts may have uniquely evoked expressions of gratitude.

Although individuals who engaged in kindness for others reported feeling more socially connected than those who engaged in open-minded behavior or kindness for themselves, they did not differ from those who acted extraverted. Participants’ prosocial acts likely involved interacting with others. This pattern of results mirrors that of a recent experiment that found that participants instructed to perform acts of kindness for others reported similar increases in feelings of social connection as those instructed to simply engage in more social interactions (Fritz et al., 2022; see also Margolis & Lyubomirsky, 2020). These results suggest that helping others is a promising way to increase momentary feelings of social connection. Indeed, people seeking to bolster their sense of connection might begin by looking for opportunities to help others.

### Limitations

Our study has several limitations. First, due to the need for multiple assessments per participant, we relied on single-item measures, limiting the reliability of the assessments. Second, although participants rated their feelings shortly after engaging in each behavior, their reports were retrospective and, thus, may have over- or underestimated their eudaimonic feelings, aligning with their post hoc theories of such feelings. Additionally, although all participants completed their assessments on days 2, 5, 9, 12, and 15, we do not know the precise lag between a particular behavior and its assessment. For example, in a day 5 assessment, a participant may have reported on an act of kindness they engaged in that morning, and another they completed 2 days prior. Third, the effect sizes were relatively small. Notably, our hypothesis tests were conservative, as all groups involved positive behaviors, which could partially explain the small group differences. However, the absence of a neutral control condition precludes addressing this possibility in the present study. Another explanation of the small effect sizes derives from a strength of our study—namely, that our participants created their own behavior change plans in accordance with their assigned activity instructions. It is possible, however, that participants engaged in behaviors that were too similar to their typical daily routines to evoke strong eudaimonic feelings. Additionally, our self-report items may not have been sensitive to the types of subjective experiences that emerged from these experimental conditions.

Finally, although we attempted to account for social desirability and demand characteristics in our analyses, we recognize that statistically controlling for these variables does not remove their causal influence on our outcomes. That is, participants may intrinsically value prosocial behavior more than the other positive behaviors induced in this study, which may have influenced their responses in ways that cannot be partialled out by including covariates in our models. It is possible, for example, that participants believed that engaging in prosocial behavior *should* be a meaningful, connecting experience, regardless of their actual experience. Future research could include relevant moderators, such as a measure of prosocial orientation, to disentangle participants’ experience of prosocial behavior from their existing preference for and orientation toward such behavior (see Canevello & Crocker, 2020, for a summary of prosocial orientation measures).

### Final Words

Previous research has explored the downstream effects of prosocial behavior on well-being and other domains, as well as the underlying psychological mechanisms involved. By contrast, we attempted here to illuminate not what prosocial behavior reaps but what it *feels* like. Our results suggest that prosociality is uniquely satisfying for the actor, resulting in greater momentary eudaimonic feelings than other positive, socially desirable behaviors.

## Supplementary Information

ESM 1 (DOCX 46 kb)

## Footnotes

1 We also tested a similar model using a 5-item composite of single-item self-report measures, excluding energy level as it is not included in all definitions of eudaimonia. Excluding energy level from the composite variable augmented the differences between conditions (see Table 3).

## Additional Information

### Data Availability

Data, materials, and R code will be available on the Open Science Foundation at https://osf.io/8zn6w/?view_only=20555aa38beb41b8b10b596f9ced5831.
