# Supplementary material for: What is Unique About Kindness? Exploring the Proximal Experience of Prosocial Acts Relative to Other Positive Behaviors
Source: Affect Sci. 2022 Oct 7;4(1):92–100. doi: 10.1007/s42761-022-00143-4 (PMC10104999; doi:10.1007/s42761-022-00143-4)
Supplement: Supplementary file 1 — (DOCX 46 kb) [file 42761_2022_143_MOESM1_ESM.docx]

**What is Unique About Kindness? Exploring the Proximal Experience of Prosocial Acts Relative to Other Positive Behaviors**

Annie Regan, Seth Margolis, Daniel J. Ozer, Eric Schwitzgebel, and Sonja Lyubomirsky, Ph.D.

University of California, Riverside

Correspondence: Annie Regan (arega008@ucr.edu)

**Supplemental Materials**

**Condition Instructions**

**Acts of kindness for the self condition:**

During the next 2 weeks, we would like you to try to do acts of kindness for yourself.

Here are some example activities to give you a better idea of what we mean by “acts of kindness for yourself”:

- Enjoying a day trip
- Treating yourself to a massage
- Having your favorite meal

Please list 5 specific plans below for where and when you will incorporate these types of activities into your daily life. For example, “When I wake up each day, I will think of one thing I could do that would involve treating myself.”

1. When _____, I will ______.
2. When _____, I will ______.
3. When _____, I will ______.
4. When _____, I will ______.
5. When _____, I will ______.

[These plans will be emailed and texted to them once, so they can refer back to them.]

**Acts of kindness for others condition:**

During the next 2 weeks, we would like you to try to acts of kindness for others.

Here are some example activities to give you a better idea of what we mean by “acts of kindness for others”:

- Cooking dinner for friends or family
- Doing a chore for a family member
- Paying for someone’s coffee in line behind you

Please list 5 specific plans below for where and when you will incorporate these types of activities into your daily life. For example, “When I see someone struggling with something, I will try to help.”

1. When _____, I will ______.
2. When _____, I will ______.
3. When _____, I will ______.
4. When _____, I will ______.
5. When _____, I will ______.

[These plans will be emailed and texted to them once, so they can refer back to them.]

**Extraversion condition:**

During the next 2 weeks, we would like you to try to act as extraverted as you can.

Here are some example activities to give you a better idea of what we mean by “extraverted”:

- Spend more time with people
- Speak up more when people say things you disagree with
- Engage in more stimulating activities

When doing these activities, try not to interact with people you already know and instead try to engage with people you don’t know very well (e.g., strangers).

Please list 5 specific plans below for where and when you will incorporate these types of activities into your daily life. For example, “When I am waiting in line for something, I will try to start conversations with people I don’t know.”

1. When _____, I will ______.
2. When _____, I will ______.
3. When _____, I will ______.
4. When _____, I will ______.
5. When _____, I will ______.

[These plans will be emailed and texted to them once, so they can refer back to them.]

**Open-mindedness condition:**

During the next 2 weeks, we would like you to try to act as open-minded as you can.

Here are some example activities to give you a better idea of what we mean by “open-minded”:

- Engage more with art, music, or literature
- Do more to pursue an intellectual curiosity
- Think more creatively, either throughout your daily life or by setting aside time to think about things in a creative manner

When doing these activities, try not to interact with people and instead try to engage with your own thoughts.

Please list 5 specific plans below for where and when you will incorporate these types of activities into your daily life. For example, “Next time I have a free half-hour, I will think creatively about a topic or piece of art that interests me.”

1. When _____, I will ______.
2. When _____, I will ______.
3. When _____, I will ______.
4. When _____, I will ______.
5. When _____, I will ______.

[These plans will be emailed and texted to them once, so they can refer back to them.]

| **Table S1** | | | |
| --- | --- | --- | --- |
| *Multilevel Model Results: Eudaimonia Composite* | | | |
| *Predictors* | *Estimates* | *CI* | *p* |
| (Intercept) | 3.49 | 3.11 – 3.87 | **<0.001** |
| Day | -0.00 | -0.01 – 0.00 | 0.831 |
| Act Number | -0.03 | -0.04 – -0.01 | **<0.001** |
| Condition: Acts of Kindness for Self | 0.17 | -0.00 – 0.35 | 0.053 |
| Condition: Extraverted Behavior | 0.14 | -0.04 – 0.32 | 0.118 |
| Condition: Open-Minded Behavior | 0.28 | 0.10 – 0.45 | **0.002** |
| Experimenter Demand | 0.12 | 0.07 – 0.17 | **<0.001** |
| Socially Desirable Responding | 0.33 | 0.26 – 0.40 | **<0.001** |
| **Random Effects** | | | |
| σ^2^ | 0.20 | | |
| τ_00_ _day:id_ | 0.45 | | |
| τ_00_ _id_ | 0.55 | | |
| ICC | 0.83 | | |
| N _day_ | 5 | | |
| N _id_ | 671 | | |
| Observations | 9183 | | |
| Marginal R^2^ / Conditional R^2^ | 0.104 / 0.850 | | |
| *Note.* Condition was coded such that a positive coefficient represents the degree to which the acts of kindness for others condition was relatively higher than each of the other conditions. | | | |

| **Table S2** | | | |
| --- | --- | --- | --- |
| *Multilevel Model Results: Meaning* | | | |
| *Predictors* | *Estimates* | *CI* | *p* |
| (Intercept) | 3.52 | 3.10 – 3.95 | **<0.001** |
| Day | 0.00 | -0.01 – 0.01 | 0.655 |
| Act Number | -0.04 | -0.05 – -0.02 | **<0.001** |
| Condition: Acts of Kindness for Self | 0.21 | 0.02 – 0.41 | **0.032** |
| Condition: Extraverted Behavior | 0.21 | 0.01 – 0.41 | **0.042** |
| Condition: Open-Minded Behavior | 0.23 | 0.03 – 0.43 | **0.023** |
| Experimenter Demand | 0.14 | 0.09 – 0.19 | **<0.001** |
| Socially Desirable Responding | 0.33 | 0.25 – 0.41 | **<0.001** |
| **Random Effects** | | | |
| σ^2^ | 0.49 | | |
| τ_00_ _day:id_ | 0.57 | | |
| τ_00_ _id_ | 0.67 | | |
| ICC | 0.72 | | |
| N _day_ | 5 | | |
| N _id_ | 671 | | |
| Observations | 9183 | | |
| Marginal R^2^ / Conditional R^2^ | 0.078 / 0.740 | | |
| *Note.* Condition was coded such that a positive coefficient represents the degree to which the acts of kindness for others condition was relatively higher than each of the other conditions. | | | |

| **Table S3** | | | |
| --- | --- | --- | --- |
| *Multilevel Model Results: Self-Confidence* | | | |
| *Predictors* | *Estimates* | *CI* | *p* |
| (Intercept) | 3.58 | 3.19 – 3.97 | **<0.001** |
| Day | -0.00 | -0.01 – 0.00 | 0.644 |
| Act Number | -0.05 | -0.07 – -0.03 | **<0.001** |
| Condition: Acts of Kindness for Self | 0.22 | 0.04 – 0.39 | **0.018** |
| Condition: Extraverted Behavior | 0.28 | 0.09 – 0.46 | **0.003** |
| Condition: Open-Minded Behavior | 0.32 | 0.13 – 0.50 | **0.001** |
| Experimenter Demand | 0.10 | 0.05 – 0.15 | **<0.001** |
| Socially Desirable Responding | 0.42 | 0.34 – 0.49 | **<0.001** |
| **Random Effects** | | | |
| σ^2^ | 0.46 | | |
| τ_00_ _day:id_ | 0.52 | | |
| τ_00_ _id_ | 0.55 | | |
| ICC | 0.70 | | |
| N _day_ | 5 | | |
| N _id_ | 671 | | |
| Observations | 9183 | | |
| Marginal R^2^ / Conditional R^2^ | 0.109 / 0.730 | | |
| *Note.* Condition was coded such that a positive coefficient represents the degree to which the acts of kindness for others condition was relatively higher than each of the other conditions. | | | |

| **Table S4** | | | |
| --- | --- | --- | --- |
| *Multilevel Model Results: Autonomy* | | | |
| *Predictors* | *Estimates* | *CI* | *p* |
| (Intercept) | 3.55 | 3.16 – 3.95 | **<0.001** |
| Day | 0.00 | -0.01 – 0.01 | 0.791 |
| Act Number | -0.02 | -0.04 – -0.00 | **0.012** |
| Condition: Acts of Kindness for Self | 0.04 | -0.14 – 0.22 | 0.655 |
| Condition: Extraverted Behavior | 0.11 | -0.07 – 0.29 | 0.238 |
| Condition: Open-Minded Behavior | 0.13 | -0.05 – 0.31 | 0.167 |
| Experimenter Demand | 0.12 | 0.07 – 0.17 | **<0.001** |
| Socially Desirable Responding | 0.33 | 0.26 – 0.40 | **<0.001** |
| **Random Effects** | | | |
| σ^2^ | 0.49 | | |
| τ_00_ _day:id_ | 0.55 | | |
| τ_00_ _id_ | 0.54 | | |
| ICC | 0.69 | | |
| N _day_ | 5 | | |
| N _id_ | 671 | | |
| Observations | 9183 | | |
| Marginal R^2^ / Conditional R^2^ | 0.073 / 0.713 | | |
| *Note.* Condition was coded such that a positive coefficient represents the degree to which the acts of kindness for others condition was relatively higher than each of the other conditions. | | | |

| **Table S5** | | | |
| --- | --- | --- | --- |
| *Multilevel Model Results: Competence* | | | |
| *Predictors* | *Estimates* | *CI* | *p* |
| (Intercept) | 3.72 | 3.34 – 4.10 | **<0.001** |
| Day | -0.00 | -0.01 – 0.00 | 0.516 |
| Act Number | -0.06 | -0.08 – -0.05 | **<0.001** |
| Condition: Acts of Kindness for Self | 0.23 | 0.06 – 0.41 | **0.009** |
| Condition: Extraverted Behavior | 0.27 | 0.09 – 0.45 | **0.003** |
| Condition: Open-Minded Behavior | 0.31 | 0.14 – 0.49 | **0.001** |
| Experimenter Demand | 0.10 | 0.05 – 0.14 | **<0.001** |
| Socially Desirable Responding | 0.39 | 0.32 – 0.46 | **<0.001** |
| **Random Effects** | | | |
| σ^2^ | 0.49 | | |
| τ_00_ _day:id_ | 0.51 | | |
| τ_00_ _id_ | 0.52 | | |
| ICC | 0.68 | | |
| N _day_ | 5 | | |
| N _id_ | 671 | | |
| Observations | 9183 | | |
| Marginal R^2^ / Conditional R^2^ | 0.101 / 0.711 | | |
| *Note.* Condition was coded such that a positive coefficient represents the degree to which the acts of kindness for others condition was relatively higher than each of the other conditions. | | | |

| **Table S6** | | | |
| --- | --- | --- | --- |
| *Multilevel Model Results: Connectedness* | | | |
| *Predictors* | *Estimates* | *CI* | *p* |
| (Intercept) | 3.50 | 3.05 – 3.96 | **<0.001** |
| Day | 0.00 | -0.00 – 0.01 | 0.164 |
| Act Number | 0.01 | -0.01 – 0.03 | 0.330 |
| Condition: Acts of Kindness for Self | 0.37 | 0.16 – 0.58 | **0.001** |
| Condition: Extraverted Behavior | 0.08 | -0.13 – 0.29 | 0.457 |
| Condition: Open-Minded Behavior | 0.52 | 0.30 – 0.73 | **<0.001** |
| Experimenter Demand | 0.12 | 0.06 – 0.17 | **<0.001** |
| Socially Desirable Responding | 0.26 | 0.18 – 0.35 | **<0.001** |
| **Random Effects** | | | |
| σ^2^ | 0.62 | | |
| τ_00_ _day:id_ | 0.61 | | |
| τ_00_ _id_ | 0.77 | | |
| ICC | 0.69 | | |
| N _day_ | 5 | | |
| N _id_ | 671 | | |
| Observations | 9183 | | |
| Marginal R^2^ / Conditional R^2^ | 0.064 / 0.710 | | |
| *Note.* Condition was coded such that a positive coefficient represents the degree to which the acts of kindness for others condition was relatively higher than each of the other conditions. | | | |

| **Table S7** | | | |
| --- | --- | --- | --- |
| *Multilevel Model Results: Energy Level* | | | |
| *Predictors* | *Estimates* | *CI* | *p* |
| (Intercept) | 3.08 | 2.54 – 3.62 | **<0.001** |
| Day | -0.01 | -0.01 – 0.00 | 0.050 |
| Act Number | 0.01 | -0.01 – 0.03 | 0.298 |
| Condition: Acts of Kindness for Self | -0.04 | -0.29 – 0.21 | 0.744 |
| Condition: Extraverted Behavior | -0.09 | -0.35 – 0.16 | 0.469 |
| Condition: Open-Minded Behavior | 0.15 | -0.10 – 0.41 | 0.247 |
| Experimenter Demand | 0.15 | 0.09 – 0.22 | **<0.001** |
| Socially Desirable Responding | 0.27 | 0.17 – 0.38 | **<0.001** |
| **Random Effects** | | | |
| σ^2^ | 0.51 | | |
| τ_00_ _day:id_ | 0.76 | | |
| τ_00_ _id_ | 1.15 | | |
| ICC | 0.79 | | |
| N _day_ | 5 | | |
| N _id_ | 671 | | |
| Observations | 9183 | | |
| Marginal R^2^ / Conditional R^2^ | 0.048 / 0.800 | | |
| *Note.* Condition was coded such that a positive coefficient represents the degree to which the acts of kindness for others condition was relatively higher than each of the other conditions. | | | |

| **Table S8** | | | |
| --- | --- | --- | --- |
| *Multilevel Model Results: Eudaimonia Composite (Excluding Energy Level)* | | | |
| *Predictors* | *Estimates* | *CI* | *p* |
| (Intercept) | 3.57 | 3.21 – 3.94 | **<0.001** |
| Day | 0.00 | -0.00 – 0.01 | 0.788 |
| Act Number | -0.03 | -0.04 – -0.02 | **<0.001** |
| Condition: Acts of Kindness for Self | 0.21 | 0.05 – 0.38 | **0.013** |
| Condition: Extraverted Behavior | 0.19 | 0.02 – 0.36 | **0.032** |
| Condition: Open-Minded Behavior | 0.30 | 0.13 – 0.47 | **0.001** |
| Experimenter Demand | 0.11 | 0.07 – 0.16 | **<0.001** |
| Socially Desirable Responding | 0.35 | 0.28 – 0.42 | **<0.001** |
| **Random Effects** | | | |
| σ^2^ | 0.22 | | |
| τ_00_ _day:id_ | 0.45 | | |
| τ_00_ _id_ | 0.51 | | |
| ICC | 0.82 | | |
| N _day_ | 5 | | |
| N _id_ | 671 | | |
| Observations | 9183 | | |
| Marginal R^2^ / Conditional R^2^ | 0.110 / 0.836 | | |
| *Note.* Condition was coded such that a positive coefficient represents the degree to which the acts of kindness for others condition was relatively higher than each of the other conditions. | | | |

| **Table S9.** *Unconditional Model: Eudaimonia Composite* | | | |
| --- | --- | --- | --- |
| *Predictors* | *Estimates* | *CI* | *p* |
| (Intercept) | 5.26 | 5.19 – 5.32 | **<0.001** |
| **Random Effects** | | | |
| σ^2^ | 0.20 | | |
| τ_00_ _day:id_ | 0.45 | | |
| τ_00_ _id_ | 0.68 | | |
| ICC | 0.85 | | |
| N _day_ | 5 | | |
| N _id_ | 671 | | |
| Observations | 9183 | | |
| Marginal R^2^ / Conditional R^2^ | 0.000 / 0.849 | | |

| **Table S10.** *Unconditional Model: Meaning* | | | |
| --- | --- | --- | --- |
| *Predictors* | *Estimates* | *CI* | *p* |
| (Intercept) | 5.34 | 5.26 – 5.41 | **<0.001** |
| **Random Effects** | | | |
| σ^2^ | 0.49 | | |
| τ_00_ _day:id_ | 0.57 | | |
| τ_00_ _id_ | 0.81 | | |
| ICC | 0.74 | | |
| N _day_ | 5 | | |
| N _id_ | 671 | | |
| Observations | 9183 | | |
| Marginal R^2^ / Conditional R^2^ | 0.000 / 0.739 | | |

| **Table S11.** *Unconditional Model: Self Confidence* | | | |
| --- | --- | --- | --- |
| *Predictors* | *Estimates* | *CI* | *p* |
| (Intercept) | 5.47 | 5.40 – 5.54 | **<0.001** |
| **Random Effects** | | | |
| σ^2^ | 0.46 | | |
| τ_00_ _day:id_ | 0.52 | | |
| τ_00_ _id_ | 0.73 | | |
| ICC | 0.73 | | |
| N _day_ | 5 | | |
| N _id_ | 671 | | |
| Observations | 9183 | | |
| Marginal R^2^ / Conditional R^2^ | 0.000 / 0.728 | | |

| **Table S12.** *Unconditional Model: Autonomy* | | | |
| --- | --- | --- | --- |
| *Predictors* | *Estimates* | *CI* | *p* |
| (Intercept) | 5.38 | 5.31 – 5.45 | **<0.001** |
| **Random Effects** | | | |
| σ^2^ | 0.49 | | |
| τ_00_ _day:id_ | 0.55 | | |
| τ_00_ _id_ | 0.67 | | |
| ICC | 0.71 | | |
| N _day_ | 5 | | |
| N _id_ | 671 | | |
| Observations | 9183 | | |
| Marginal R^2^ / Conditional R^2^ | 0.000 / 0.713 | | |

| **Table S13.** *Unconditional Model: Competence* | | | |
| --- | --- | --- | --- |
| *Predictors* | *Estimates* | *CI* | *p* |
| (Intercept) | 5.48 | 5.41 – 5.55 | **<0.001** |
| **Random Effects** | | | |
| σ^2^ | 0.49 | | |
| τ_00_ _day:id_ | 0.51 | | |
| τ_00_ _id_ | 0.68 | | |
| ICC | 0.71 | | |
| N _day_ | 5 | | |
| N _id_ | 671 | | |
| Observations | 9183 | | |
| Marginal R^2^ / Conditional R^2^ | 0.000 / 0.708 | | |

| **Table S14.** *Unconditional Model: Connectedness* | | | |
| --- | --- | --- | --- |
| *Predictors* | *Estimates* | *CI* | *p* |
| (Intercept) | 4.98 | 4.90 – 5.06 | **<0.001** |
| **Random Effects** | | | |
| σ^2^ | 0.62 | | |
| τ_00_ _day:id_ | 0.61 | | |
| τ_00_ _id_ | 0.90 | | |
| ICC | 0.71 | | |
| N _day_ | 5 | | |
| N _id_ | 671 | | |
| Observations | 9183 | | |
| Marginal R^2^ / Conditional R^2^ | 0.000 / 0.710 | | |

| **Table S15.** *Unconditional Model: Energy Level* | | | |
| --- | --- | --- | --- |
| *Predictors* | *Estimates* | *CI* | *p* |
| (Intercept) | 4.89 | 4.80 – 4.98 | **<0.001** |
| **Random Effects** | | | |
| σ^2^ | 0.51 | | |
| τ_00_ _day:id_ | 0.76 | | |
| τ_00_ _id_ | 1.27 | | |
| ICC | 0.80 | | |
| N _day_ | 5 | | |
| N _id_ | 671 | | |
| Observations | 9183 | | |
| Marginal R^2^ / Conditional R^2^ | 0.000 / 0.800 | | |

| **Table S16.** *Unconditional Model: Eudaimonia Excluding Energy Level* | | | |
| --- | --- | --- | --- |
| *Predictors* | *Estimates* | *CI* | *p* |
| (Intercept) | 5.33 | 5.26 – 5.40 | **<0.001** |
| **Random Effects** | | | |
| σ^2^ | 0.22 | | |
| τ_00_ _day:id_ | 0.45 | | |
| τ_00_ _id_ | 0.65 | | |
| ICC | 0.83 | | |
| N _day_ | 5 | | |
| N _id_ | 671 | | |
| Observations | 9183 | | |
| Marginal R^2^ / Conditional R^2^ | 0.000 / 0.834 | | |
